# Nisin Purification from a Cell-Free Supernatant by Electrodialysis in a Circular Economy Framework

**DOI:** 10.3390/membranes14010002

**Published:** 2023-12-21

**Authors:** Alexandre Rulence, Véronique Perreault, Jacinthe Thibodeau, Loubna Firdaous, Ismail Fliss, Laurent Bazinet

**Affiliations:** 1 UMR Transfrontalière BioEcoAgro N°1158, Lille University, Institut National de Recherche pour l’Agriculture, l’Alimentation et l’Environnement (INRAE), Liège University, Université de Picardie Jules Verne (UPJV), YNCREA, Artois University, Littoral Côte d’Opale University, ICV—Institut Charles Viollette, F-59000 Lille, France; alexandre.rulence.1@ulaval.ca (A.R.); loubna.firdaous@univ-lille.fr (L.F.); 2 Institute of Nutrition and Functional Foods (INAF), Dairy Research Center (STELA), Laval University, Quebec, QC G1V 0A6, Canada; veronique.perreault.5@ulaval.ca (V.P.); jacinthe.thibodeau.1@ulaval.ca (J.T.); ismail.fliss@fsaa.ulaval.ca (I.F.); 3 Laboratoire de Transformation Alimentaire et Procédés ÉlectroMembranaires (LTAPEM, Laboratory of Food Processing and Electro-Membrane Processes), Food Science, Laval University, Quebec, QC G1V 0A6, Canada

**Keywords:** electrodialysis, nisin purification, circular economy, antimicrobial activity

## Abstract

Nisin, an antimicrobial peptide produced by *Lactococcus lactis* strains, is a promising natural preservative for the food industry and an alternative to antibiotics for the pharmaceutical industry against Gram-positive bacteria. Nisin purification is commonly performed using salting out and chromatographic techniques, which are characterized by their low yields, the use of solvents and the production of large volumes of effluents. In the present work, the purification of nisin from a cell-free supernatant (CFS), after the production of nisin by fermentation on a whey permeate medium, was studied using ammonium sulfate precipitation and electrodialysis (ED) as a promising eco-friendly process for nisin purification. Results showed an increase in nisin precipitation using a 40% ammonium sulfate saturation (ASS) level with a purification fold of 73.8 compared with 34.5 and no purification fold for a 60% and 20% ASS level, respectively. The results regarding nisin purification using ED showed an increase in nisin purification and concentration fold, respectively, of 21.8 and 156 when comparing the final product to the initial CFS. Nisin-specific activity increased from 75.9 ± 4.4 to 1652.7 ± 236.8 AU/mg of protein. These results demonstrated the effectiveness of ED coupled with salting out for nisin purification compared with common techniques. Furthermore, the process was noteworthy for its relevance in a circular economy scheme, as it does not require any solvents and avoids generating polluting effluents. It can be employed for the purification of nisin and the recovery of salts from salting out, facilitating their reuse in a circular economy.

## 1. Introduction

Nisin is a naturally occurring antimicrobial peptide (AMP) that is produced by lactic acid bacteria (LAB) and was first commercialized in 1950 for the inhibition of pathogenic strains of Clostridium. It has since been studied extensively and used for biological preservation applications and medical treatments. Its properties, such as high thermal stability at low pH [1,2], great solubility in water and the fact that it is colourless and odourless [3], make nisin a high valuable molecule. It is also the most studied bacteriocin for food applications as a natural preservative [4,5,6], with a market size estimated at USD 443 million and projected to reach USD 553 million by 2025 [7]. Indeed, nisin is actually the only bacteriocin recognized as Generally Recognized As Safe (GRAS) by the FDA (U.S. Food and Drug Administration, Washington, DC, USA) [8].

However, nisin is generally produced using a rich and complex liquid culture, such as de Man, Rogosa and Sharp (MRS), one of the main broths used for industrial production, hampering its yields and further purification [9]. Numerous alternatives were studied to improve nisin production, such as using agro-industrial by-products as cheap broths. Whey permeate produced after ultrafiltration of whey is actually the most commonly used by-product for bacteriocin and nisin production [10,11]. Nisin production is then followed by downstream processing for its further but partial separation and purification from the initial culture medium. Hence, nisin is actually commercialized as products containing 0.5 to 5% of nisin [12]. Nisaplin^TM^ by Danisco is one of the historical forms of commercial nisin, containing up to 2.5% of nisin and composed of up to 80% (*w*/*v*) NaCl salts, around 2 to 5% of denatured milk proteins and carbohydrates issued from the initial culture medium [13,14].

Several methods have been reported in the literature regarding nisin purification such as cell adsorption [15], membrane separation [16,17] and solvent extraction [18]. However, these methods display a high cost and complex applications at larger scale and introduce compounds of regulatory concern and large volumes of effluents that make them not suitable for widespread nisin applications [19]. The purification of nisin by salting out is also another method reported in the literature [20,21]. Ammonium sulfate and sodium chloride are the most often used salts for nisin precipitation after culture media clarification (removing the cell) into a cell-free supernatant (CFS) [22]. They are generally applied before chromatographic purification techniques such as expanded bed ion exchange, immunoaffinity chromatography or hydrophobic interaction chromatography [9,23,24]. Salting out is based on protein solubility in water, where proteins need a low concentration of salt ions in order to remain soluble in a solution [20,24,25,26]. The use of salting out for nisin purification was reported in multiple studies which used sodium chloride [27,28,29,30] and ammonium sulfate precipitation at different optimal salt saturation levels for nisin recovery [20,24,25,26,31]. However, one of the main hurdles of salting out processes are the generation of significant amounts of saline effluents which represent an ecological and economical issue regarding their further treatment and disposal [32].

Recently, the purification of nisin from a commercial solution was studied using conventional electrodialysis (ED) [33]. Conventional ED, an electrochemical method using electrodes and ion exchange membranes (IEM), allows for the purification and demineralization of charged molecules under the application of an electric potential gradient [34]. ED is a cost-effective process, commonly used for desalination of water and wastewater, since it has a low energy consumption compared with thermal processes (evaporation/distillation) and reverse osmosis [35,36]. Moreover, the main application of ED in the food industry is demineralization of whey [37,38]. In a previous study from Rulence et al. [33], the effectiveness of ED for the demineralization and purification of nisin in comparison with a multiple-step centrifugation process that had already been used was demonstrated; a purification factor of 1.19, fold concentration of 10.39 and protein recovery yield of 83.8% were achieved. The demineralization and purification by ED of high salt content products thus represent an interesting way to purify nisin without the production of polluting saline effluents. However, to the best of our knowledge, there are no studies reporting on the implementation and the use of electromembrane technology for nisin purification from a food by-product cell-free supernatant.

In this context, the present study aimed to demonstrate the feasibility of nisin purification from a complex cell-free supernatant produced from a whey permeate culture medium using ammonium sulfate salting out and electrodialysis. The specific objectives were to (1) determine the optimal ammonium sulfate salting out concentration for nisin pre-concentration, (2) purify nisin by ED, (3) evaluate the ED performances and potential fouling of membranes and (4) characterize the antimicrobial activity and concentration factor of the final nisin.

## 2. Materials and Methods

### 2.1. Materials

Na_2_SO_4_, used as electrode rinsing solution, and KCl, used as concentrate for the purification of nisin by ED, were purchased from BDH (VWR International Inc., Mississauga, ON, Canada), while HCl, NaOH and (NH_4_)_2_SO_4_ (ammonium sulfate) were from Fisher Scientific (Montréal, QC, Canada). LC-MS-grade water, acetonitrile and formic acid were purchased from Fischer Scientific (Ottawa, ON, Canada). Pierce^®^ BCA Protein Assay Kit was also purchased from Fischer Scientific (Ottawa, ON, Canada). Whey permeate powder was provided by Agropur (Québec, QC, Canada).

### 2.2. Production of Nisin Cell-Free Supernatant (CFS)

Nisin was produced by fermentation with *L. lactis* UL 719 as the producer strain in a supplemented whey permeate powder culture broth based on previous works [39,40]. Whey permeate powder was solubilized at 10% (*w*/*v*) and supplemented with 1.5% (*v*/*v*) of Tween 80 in 1600 mL of distilled water. The whey permeate solution was adjusted to pH 3.5. For whey permeate supplementation, a 400 mL solution of yeast extract at 5 g/L was prepared alongside. The solutions were then sterilized at 121 °C in separate flasks. The supplemented whey permeate solution was adjusted at pH 6.8 before fermentation using 4 M NaOH in sterile distilled water. The final concentration of the medium was 8% (*w*/*v*) whey permeate, 4 g/L yeast extract and 0.1% (*v*/*v*) Tween 80.

The supplemented whey permeate medium prepared previously was then inoculated with an overnight grown culture of *L. lactis* UL 719 (MRS, 30 °C), corresponding to an inoculation of 1% (*v*/*v*). The inoculated medium was then incubated at 30 °C for 24 h under low agitation. After fermentation, biomass was removed by centrifugation at 3400× *g* for 15 min at 4 °C in order to recover the cell-free supernatant (CFS).

### 2.3. Determination of Optimal Ammonium Sulfate Salting Out Concentration

In order to determine ammonium sulfate saturation (ASS) level, pre-tests were conducted on small volumes of produced CFS. Three different ASS levels were tested: 20, 40 and 60%. These ASS levels of ammonium sulfate salts were chosen according to results already reported in the literature in terms of nisin purification and yields [20,26,31]. A volume of 2 L of CFS containing nisin was produced by fermentation of *L. lactis* UL 719 in whey culture medium as described earlier and was aliquoted into three solutions with a 200 mL volume disposed in 500 mL Erlenmeyer flasks. Prior to the addition of ammonium sulfate, the CFS was adjusted at pH 3.8 using 1 M HCl solution to perform the salting out at optimal pH conditions regarding nisin stability [8]. Ammonium sulfate was progressively added to each flask, and the salted CFSs were then cooled at 4 °C under low magnetic agitation for 3 h. After precipitation, solutions were centrifuged at 9600× *g* for 60 min at 4 °C to recover pellets containing proteins, including nisin [20,21]. Pellets were then resolubilized in 50 mL of distilled water. The solutions were tested for their antimicrobial activity and protein content. The experiments were carried out in triplicate for each tested ASS level.

### 2.4. Purification by ED

**Figure 1:**
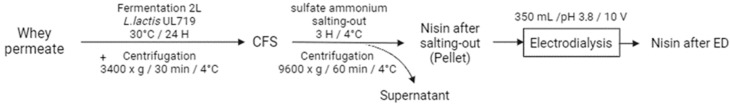
Flowchart of nisin production and purification from a cell-free supernatant (CFS) using ammonium sulfate salting out and electrodialysis (ED).

Nisin purification from the CFS was tested using ED process, as shown in Figure 1. Nisin was produced in 2 L of whey permeate medium and the salting out procedure was carried out at the previous optimal ammonium sulfate salt concentration saturation level. The solution was then centrifuged at 9600× *g* for 60 min at 4 °C. The pellet was then solubilized in 30 mL of distilled water and freeze-dried. This solution was identified as ‘’nisin after salting-out” (Figure 1).

**Figure 2:**
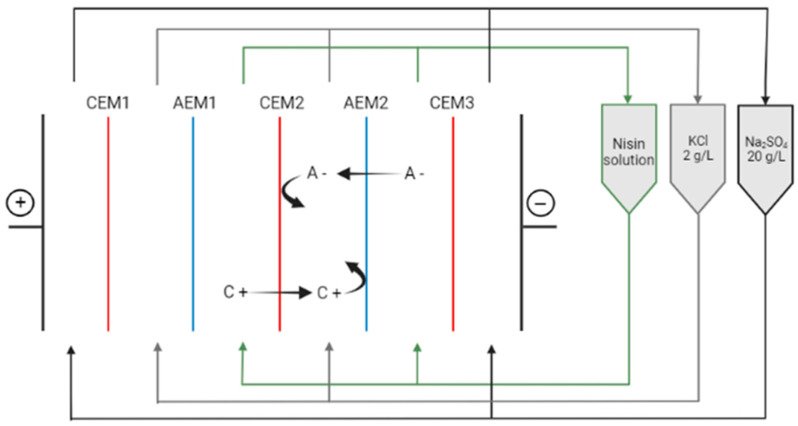
Configuration of the electrodialysis cell. CEM: cation exchange membrane; AEM: anion exchange membrane. C+: cations, A−: anions.

The ED cell was an MP-type cell (ElectroCell AB, Täby, Sweden), as described in [33] and presented in Figure 2. The ED stack was composed of food-grade Neosepta ion exchange membranes (AMX-fg and CMX-fg) purchased from Astom (Tokyo, Japan). Prior to ED, the pellet obtained after salting out (nisin after salting out) was solubilized in 350 mL of distilled water, and the pH was adjusted to 3.8 using 1 M HCl and agitated overnight at 4 °C. ED parameters were established according to [33] such as the voltage maintained at 10 V and the pH of the nisin solution controlled at pH 3.8 with a HCl 1 M solution throughout the process to avoid decrease in nisin activity. Samples were taken every 15 min to assess nisin antimicrobial activity. Conductivity and pH were also monitored throughout the process. Purification of nisin by ED was stopped when the solution containing nisin reached a 95% demineralization rate. The product after ED was then freeze-dried for further HPLC analyses.

### 2.5. Analyses

#### 2.5.1. Nisin Antimicrobial Activity Bioassay

Nisin antimicrobial activity was determined by critical dilution assay [41] in the initial CFS after salting out and after ED. It was performed by two-fold diluted samples as serial dilution in 125 µL of Tryptic soy broth (TSB) medium in a 96-well Falcon microtiter plate. *L. ivanovii* HPB28 was used as a sensitive strain cultured overnight at 37 °C in TSB medium [41]. Culture was diluted 100-fold before inoculation by addition of 50 µL of diluted samples. After inoculation, the microplates were incubated at 37 °C for 18 to 24 h, and the nisin activity (AU/mL) was assessed using Equation (1) by reading the absorbance at a wavelength of 630 nm.

(1) $${Nisin\;activity\;(AU/mL}^{-1})=\frac{1000}{125}\times \frac{1}{d}$$

where *d* is the highest dilution that completely prevented growth of the tested organism after incubation.

#### 2.5.2. Protein Content

For optimal ammonium sulfate concentration, protein content was assessed. Prior to protein content determination, the samples were demineralized using Spectra/Por^®^ 6 Dialysis Membranes, Regenerated CelluloseMWCO 1 kDa (Spectrum^®^ Laboratories, Compton, CA, USA). Total protein quantification was then assessed using the bicinchoninic acid (BCA) protein assay from Thermo Fischer scientific (Pierce^®^ BCA Protein Assay Kit, Burlington, ON, Canada). Standard curves were performed using bovine serum albumin (BSA) solutions ranging from 25 to 2000 µg/mL. Assays were performed in a 96-well Falcon microtiter plate and the absorbance read at 562 nm with a microplate spectrophotometer (Agilent BioTek PowerWave HT, Santa Clara, CA, USA) [42].

For ED purification, protein content was determined on freeze-dried samples from CFS, nisin after salting out and nisin after ED using a Rapid Micro N Cube (Elementar, Francfort-sur-le-Main, Germany) based on the micro-Dumas combustion method. Nitrogen content was converted into protein content on a dry basis using a conversion factor of 6.25 [30].

#### 2.5.3. Antimicrobial-Specific Activity

Nisin-specific activity was calculated for each ammonium sulfate concentration tested in the ammonium sulfate concentration determination protocol as well as during the ED purification protocol for the CFS, nisin after salting out and nisin after ED. Antimicrobial yield activity and specific activity were calculated using total antimicrobial activity and total protein content determined for each sample by using Equations (2) and (3):

(2) $$\mathrm{Antimicrobial}\;\mathrm{activity}\;\mathrm{yield}\;(\%)\;=\;\frac{Total\;nisin\;activity\;of\;sample\;(AU)}{Total\;nisin\;activity\;initial\;(AU)}\times 100$$

(3) $$Specific\;activity\left({AU/mg}^{-1}\right)=\frac{Total\;activity\;(AU)}{Total\;protein\;(mg)}$$

Assessing specific activity for each sample, a fold purification was determined using Equation (4):

(4) $$\mathrm{Fold}\;\mathrm{purification}=\frac{Specific\;activity\left(AU/mg\;of\;protein\right)\;of\;sample}{Specific\;activity\left(AU/mg\;of\;protein\right)\;of\;initial\;solution}$$

#### 2.5.4. Conductivity

A YSI conductivity meter model 3100 (cell constant k = 1 cm^−^^1^) (Yellow Springs Instruments Co., Yellow Springs, OH, USA) was used to measure the conductivity of the salt precipitated samples and to monitor the conductivity of nisin and KCl solutions during ED. Conductivity monitoring was also carried out to follow the demineralization of the nisin solution during ED, using Equation (5):

(5) $$Demineralization\;rate\left(\%\right)=\left(1-\frac{Current\;conductivity}{Initial\;conductivity}\right)\times 100$$

#### 2.5.5. pH

The pH of the solutions before and after salting out as well as solutions of nisin and KCl during ED were measured with a VWR Symphony pH-meter SP20 Thermo Orion (West Chester, PA, USA).

#### 2.5.6. Membrane Characterization

Membrane thickness and electrical conductivity were measured for each membrane before and after ED treatments of the nisin solution to evaluate their potential fouling. Prior to analyses, membranes were equilibrated in 0.5 M NaCl solution for 30 min.

Membrane thickness was measured using an electronic digital micrometer of 10 mm diameter flat contact point (Marathon watch company LTD, Richmond Hill, ON, Canada) at six different locations on the membrane.

Membrane conductance was measured using a YSI conductivity meter model 3100 Yellow Springs Instruments Co. (Yellow Springs, OH, USA) equipped with a specially designed clip from the Laboratoire des Matériaux Echangeurs d’Ions (Université Paris XII, Créteil, Val de Marne, France). The membrane conductance of the effective membrane surface was taken in the reference solution and at six different locations. The membrane electrical resistance was then calculated as described by Lteif et al. (1999) and Lebrun et al. (2003) [43,44], according to Equation (6):

(6) $$R_{m}=\frac{1}{G_{m}}=\frac{1}{G_{m+S}}-\frac{1}{G_{S}}=R_{m+S}-R_{S}$$

where R_m_ and *G_m_* are the transverse electric resistance (Ω) and conductance (S) of the membrane, respectively. *R_s_* and *G_s_* are the resistance (Ω) and conductance (S) of the reference solution, while *R_m+s_* and *G_m+s_* are the resistance (Ω) and conductance (S) of the membrane and the reference solution, respectively. Membrane conductivity was calculated according to Equation (7):

(7) $$\kappa =\frac{L}{R_{m}A}$$

where κ is the membrane electrical conductivity (S/cm), *L* the thickness of the membrane (cm) and *A* the electrode area (1 cm^2^).

#### 2.5.7. ED Energy Consumption (EC)

Energy consumption was calculated according to Equation (8) [45]:

(8) $$EC\left(Wh\right)=U\int_{o}^{t}I(t)\;dt$$

where U is the voltage applied (V), I is the intensity (A), and t is the duration of the process (s). The EC was expressed in Wh.

#### 2.5.8. UPLC-MS Analysis for Nisin Concentration Factor Determination

Nisin fold concentration was determined with freeze-dried nisin samples at 1% (*w*/*v*) on a powder basis and filtered through 0.22 µm filters on nisin after salting out and nisin after ED samples. Nisin was separated and identified using UPLC coupled with Q-TOF system. The samples were analyzed using a 1290 Infinity II UPLC (Agilent Technologies, Santa Clara, CA, USA), which was composed of a binary pump (G7120A), a multisampler (G7167B), an in-line degasser and a variable wavelength detector (VWD G7114B) adjusted to 214 nm. A gradient elution of the mobile phase consisting of solvent A (LC-MS-grade water with 0.1% formic acid) and solvent B (LC-MS-grade acetonitrile with 0.1% formic acid) was applied at a constant temperature of 45 °C, with solvent B increasing from 3% to 22% in 15 min, increasing to 28% until 20 min, and again, to 45% until 25 min and finally ramping to 95% in order to wash the column for 3 min before returning to initial conditions for equilibration prior to the following injection. Using a hybrid ion mobility quadrupole time-of-flight (IM-Q-TOF, Agilent Technologies, Santa Clara, CA, USA), the determination of the accurate mass was performed. Nitrogen was selected as the drying (13.0 L/min, 150 °C) and nebulizer gas (30 psi). The voltages were set at 400 V for the fragmentor, 300 V for the nozzle and 3500 V for the capillary. Positive mode at Extended Dynamic Range, 2 Ghz, 3200 m/z was used to record signals. A volume of 20 µL of each prepared sample was loaded onto a Poroshell 120 EC-C18 column (2.1 × 100 mm i.d., 2.7 micron, Agilent, Santa Clara, USA) at a flow rate of 400 µL/min. Acquisition of data and analysis were performed using the Agilent Mass Hunter Software package (LC/MS Data Acquisition, Version B.09.00 and Qualitative Analysis for IM-MS, Version B.07.00 Service Pack 2 with BioConfirm Software).

These analyses were carried out to assess nisin concentration factor using the UV chromatogram nisin peak area. For each solution, the nisin peak was determined by mass spectrometry, using 3500 Da as molecular weight reference for nisin [2,46].

The determination of the nisin peak and its area allowed nisin purity comparison of each generated fraction by calculating a concentration factor according to the nisin area in the initial solution (CFS) using Equation (9):

(9) $$\mathrm{Concentration}\;\mathrm{factor}\;=\frac{Nisin\;peak\;area\left(AU\right)\;sample}{Nisin\;peak\;area\left(AU\right)\;initial\;nisin\;solution\;(CFS)}$$

#### 2.5.9. Statistical Analyses

Data obtained from optimal ammonium sulfate concentration determination and electrodialysis purification protocols were reported as mean values ± standard deviations. Each experiment was performed in triplicate. A Student’s *t*-test (*p* < 0.05) was performed to determine statistical difference for membrane characterization. For the different parameters measured during the ED protocol, one-way ANOVA (*p* < 0.05) were performed. SigmaPlot software (version 12.0, Systat Software, San Jose, CA, USA) was used to perform statistical analyses.

## 3. Results and Discussion

### 3.1. Optimization of Ammonium Sulfate Salting Out

Before the nisin purification with ED, different ASS levels for nisin precipitation were tested to determine the optimal one, since different ASS levels were reported in the literature [20,24,26]. For the initial CFS, a specific activity of 72.0 ± 12.7 AU/mg of protein was found. Compared with 20 and 60% ASS, a 40% ASS produced the nisin solution with the highest specific activity (*p* < 0.05): a specific activity of 5310.4 ± 1007.1 AU/mg of protein (Table 1). A level of 60% salt saturation showed a specific activity of 2481.6 ± 827.2 AU/mg of protein, while at a 20% salt saturation level, no specific activity was observed, since no pellet could be recovered after salting out and centrifugation. Using the specific activity of 40 and 60% salt saturation fractions and Equation (4), a fold purification of 73.8 and 34.5 were determined when compared with the CFS specific activity. No purification fold could be determined for 20%, since the protein content was below the threshold of the BSA standards (25 µg/mL) in the BCA method. This confirmed the absence of pellets after salting out and centrifugation. Using Equation (2), a nisin antimicrobial activity yield of 60 and 50% for the salting out at 40% and 60%, respectively, was found. No activity yield could be determined for the salting out at a 20% saturation level.

Those results are in accordance with the ones found by Tafreshi et al. [20] and Burianek et al. [26], who determined the highest nisin purification rate at 40% of ASS concentration with a comparable specific activity of 2000–6000 AU/mg of protein after salting out on an MRS broth [26]. However, the fold purification obtained by salting out with 40% ammonium sulfate in Tafreshi et al. [20] was superior, with a fold purification of 168.8 and a 90% activity yield compared with 73.8 with a 60% activity yield in the present study (Table 1). This could be explained by the different compositions of the media, since Tafreshi et al. worked on an MRS broth compared with a supplemented whey permeate here. It could also be due to the difference in the precipitation duration applied on CFS containing nisin, since Tafreshi et al. applied a longer time for salt precipitation (24 h compared with 3 h in this study). However, other studies have reported different ASS levels for nisin purification with lower fold purification such as Choi et al. [31] (nisin fold purification of 5 using 35% ASS concentration) or Lee et al. (2002) [25] (nisin fold purification of 3.8 at 50% of ASS concentration). Another study demonstrated a fold purification of 2.5 using ammonium sulfate at 60–80% [24]. Once again, the differences in nisin purification fold using ammonium sulfate could be explained by the difference in composition of the media containing nisin (MRS vs. whey permeate) and the duration of the salting out process. Indeed, the mineral environment of the protein is of importance, since the success of the protein salting out process depends on its hydrophobicity and hydrophilicity [47]. To be soluble, proteins need to interact with water molecules through their hydrophilic regions and to form a hydration barrier. Protein solubility is therefore correlated with the accessibility of water molecules in order to solvate the proteins. The addition of salts in a protein solution increases the competition between protein and salts to access water molecules, with salts interacting with water molecules to be solubilized. A large addition of salts in a protein solution favours salt interactions with water, thereby hindering proteins’ access to water molecules and reducing their hydration barrier. Proteins lose their solubility and precipitate in aggregate, interacting through their hydrophobic regions [47,48,49]. The observed differences in the present study, compared with the data reported in the literature could, therefore, depend on multiple factors such as the nature of the different proteins that are present in the medium and the overall composition of the medium. The exposure duration of the protein to the salts is also an important factor, since a longer duration leads to greater binding of salts to the proteins to be able to precipitate [21]. This could explain the difference observed between the three different ASS levels used for the salting out process, with a higher purification fold observed with 40% ASS level. Another reason for finding less nisin in the pellet at a 60% ASS level could be the formation of floating pellets due to the high concentration, as reported in the literature [28]. The differences with the literature could mostly be explained by the difference in the medium composition, as discussed above. Whey permeate contains a low protein content [50] compared with MRS broth, having a high concentration of meat and yeast extract proteins.

### 3.2. Purification of Nisin by Electrodialysis

For nisin purification using ED, a salting out at 40% ASS was performed, as determined in the previous part, on 2000 mL of CFS produced after 24 h of fermentation on a supplemented whey permeate.

#### 3.2.1. Evolution of Demineralization Rate, pH and Antimicrobial Activity

**Figure 3:**
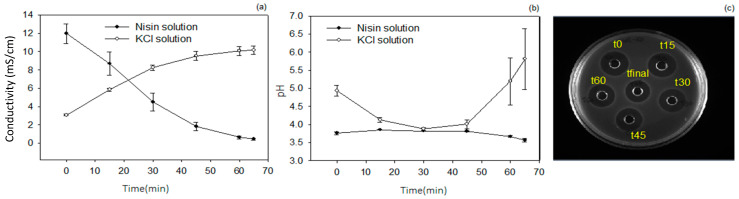
Evolution of (**a**) nisin and KCl solution conductivity, (**b**) pH of nisin and KCl solution and (**c**) antimicrobial activity of the nisin solution during demineralization process. (●) nisin solution and (○) KCl solution.

Before ED, the conductivity of the nisin solution was 12.0 ± 1.1 mS/cm, and it decreased up to 0.4 ± 0.1 mS/cm at the end of the ED process (Figure 3a), corresponding to a 96.6 ± 0.7% demineralization rate. In parallel, as expected, the conductivity of the KCl solution increased during the process, reaching a final conductivity of 10.2 ± 0.5 mS/cm and demonstrating the migration of salts from the nisin compartment to the KCl one. The pH of the nisin solution was maintained at 3.8 throughout the ED process (Figure 3b) in order to preserve the nisin’s structural stability and antimicrobial activity [33], as confirmed in Figure 3c. The pH of the KCl solution decreased from 4.9 to 3.8 in the first 30 min of demineralization, and then increased until the end of the process, from 3.8 to 5.8. The decrease in pH in the first 30 min was mainly due to the migration of H^+^ that were present in the nisin solution to the KCl solution, while the increase in pH after 30 min of demineralization can be explained by the decrease in conductivity of the nisin solution (close to 70% demineralization). Indeed, such a depletion of ions in the nisin solution induced water molecule dissociation at the cation exchange membrane interface (CEM) between the nisin and KCl solution compartment [33] (Figure 2). Water dissociation occurs when the limiting current density (LCD) is reached, i.e., the LCD corresponding to the moment where the concentration of ionic species at one of the membrane interfaces is almost zero [51]. Water dissociation could therefore explain the pH increase in the KCl solution, OH^−^ ions being produced in the nisin solution at a higher concentration than H^+^ ions migrating through the CEM membranes to the KCl solution. Nevertheless, the increase in pH in the KCl solution did not limit the purification of the nisin solution. These results are therefore comparable to those found in a previous work on a commercial nisin solution [33], since the nisin solution conductivity presented the same evolution and allowed us to maintain nisin activity when under pH control throughout the process. The difference in duration required to reach 95% demineralization (65 vs. 75 min) could be explained by the difference in the initial nisin solution conductivity (12.0 vs. 27.9 mS/cm in the previous study). The conductivity evolution was also comparable to the ones described in the literature when low conductivity was accompanied by an increase in pH [52].

#### 3.2.2. Membrane Characterization

The measurements of the ion exchange membrane electrical conductivity and thickness were performed before and after ED in order to identify the presence of potential fouling that could occur and affect their integrity. No difference was observed for each membrane regarding their thickness and conductivity (*p* > 0.05; Table 2), with thickness values ranging from 0.138 to 0.145 mm and conductivity values ranging from 7.73 to 8.47 mS/cm for CEM and from 5.23 to 5.90 mS/cm for AEM.

The similarities of membrane characteristics in terms of thickness and conductivity before and after ED validated the absence of fouling. This was confirmed visually and also by touching the membrane. This is in accordance with results found in a previous work [33], where no fouling was observed after demineralization by ED of a commercial nisin solution when the pH was maintained at a constant value of 3.8.

#### 3.2.3. Energy Consumption

The EC of the ED regarding nisin demineralization was found to be 5.6 ± 0.6 Wh (energy required to treat the 350 mL nisin solution) or 16.3 kWh/m^3^. When compared with the literature, this value was lower than that reported by Khetsomphou et al. [53] for ED whey demineralization using a CMX membrane (13.31 ± 0.24 Wh). However, the value of 16.3 kWh/m^3^ was comparable to the 17 kWh/m^3^ reported by Kress (2019) [54] for the desalination of seawater by ED. Indeed, the solution conductivity is of importance, since a higher energy consumption is reported for solutions with high conductivity compared with solutions with lower conductivity [55]. The energy consumption calculated could also be overestimated, since ED was performed in batch mode at a small scale. Nonetheless, ED represents a process characterized by a lower energy consumption when applied to demineralization [35,36].

#### 3.2.4. Nisin Final Purification

The purification of nisin from CFS by salting out and demineralization by ED increased the nisin specific activity from 75.9 ± 4.4 to 1652.7 ± 236.8 AU/mg of protein, corresponding to a fold purification of 21.8 (Table 3). This corresponded to a 70% nisin activity recovery yield by dividing the nisin total activity after ED with the nisin total activity in the initial CFS. No protein content could be determined after the salting out step due to the very limited quantity of samples remaining. The purification fold obtained after coupling ED and salting out was lower than the one obtained with the previous salting out protocol assessing the optimal ASS level: 21.8 with 70% nisin activity vs. 73.8 with 60% nisin activity yield (Table 1). This difference in purification fold and activity yield could be due to the difference in scale regarding salting out, where the ASS level determination protocol was conducted on 200 mL of CFS compared with the 2000 mL of CFS for the salting out followed by ED.

**Figure 4:**
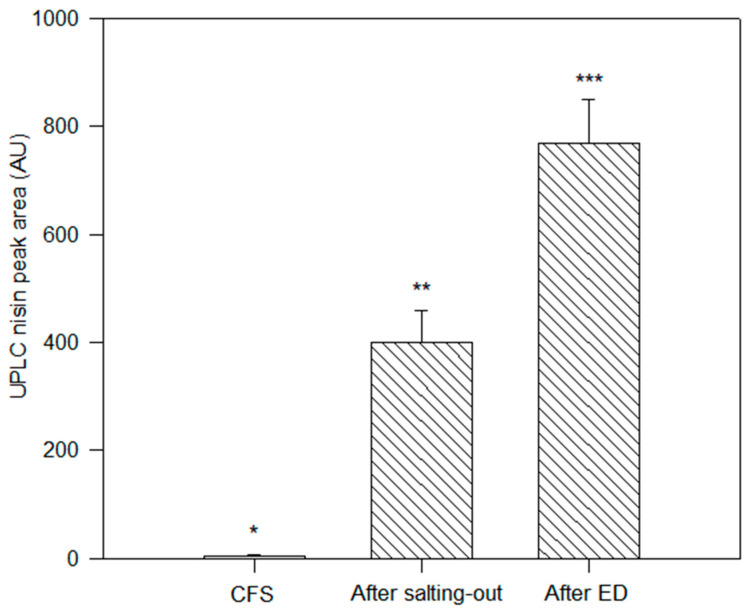
UPLC peak area of nisin in the initial CFS, after salting out and after ED purification. Nisin peak was confirmed by MS according to its 3500 Da molecular weight. Columns with a different number of asterisks (*) are significantly different at *p* < 0.05 (*t*-test).

These freeze-dried samples (CFS, nisin after salting out and nisin after ED) were further analyzed by UPLC at 1% (*w*/*v*) on a powder basis for the determination of the concentration fold at the different steps of the process (Figure 4). The nisin peak after ED presented an area under the curve of 770.1 ± 79.9 AU, which was significantly higher than the one found for the initial CFS solution of 4.9 ± 0.7 AU (*p* < 0.05). The nisin peak after salting out has an area under the curve of 399.6 ± 59.3 AU, which is significantly higher than the one of CFS but lower than the nisin peak after ED. Those nisin peak areas, compared with the initial CFS peak area, correspond to concentration factors of 80- and 156-fold, respectively, for salting out and ED.

In comparison with our previous work [33], where nisin purification from a commercial solution was performed by ED, and compared with a multistep centrifugation, fold purifications of 1.2 and 6.2 with protein yields of 83.8 and 38.5% were found for ED and centrifugation, respectively. These techniques also displayed great concentration factors of 10.4 for ED and 47.8 for the multistep centrifugation process. However, despite its great purification and concentration factor, the multistep centrifugation process is not applicable at an industrial scale for nisin purification due to the large production of saline effluents. The application of ED for the purification of nisin from a CFS compared with a nisin from a commercial solution is of greater interest: a purification fold of 21.8 compared with 1.2 and a concentration fold of 158 compared with 10.4 [33].

The results obtained can, moreover, be compared with other common techniques used for nisin purification (Table 4). For ammonium sulfate precipitation, lower purification folds ranging from 2.3 to 5.1 were obtained, with nisin yields ranging from 62 to 98% [24,25,31]. Tafreshi et al. (2020) [20] demonstrated a purification fold of 168.8 with a 90% activity yield using an ammonium sulfate precipitation. As explained previously, the difference in purification folds could mostly be explained by the difference in the media compositions, since the works cited used MRS broths to produce nisin and perform ammonium sulfate salting out. The present results were also comparable to other common techniques used for nisin purification such as chromatographic techniques. Cation exchange chromatography demonstrated a 31-fold purification (compared with 21.8), with a yield of 20% (compared with 70%) [20], while immunoaffinity chromatography demonstrated nisin purification up to 10-fold, with a 72% nisin activity recovery [23]. Expanded bed ion exchange chromatography demonstrated a greater purification fold of 31, with a 90% yield of nisin from a CFS [9]. Solvent extraction techniques were also used for nisin purification. Methanol and ethanol extractions were applied on a 2.5% nisin commercial solution and showed purification factors of 5.3 and 5.5, with activity yields of 91 and 85%, respectively [56]. Similar results were reported by Taylor et al., who used the same solvents and a commercial nisin solution, showing purification factors of 5.98 and 1.93 with yields of 52.4 and 63.0%, respectively, for methanol and ethanol [57]. Chloroform extraction was also studied on nisin purification, with a purification factor of 37.4 and a 24% yield [20]. Less conventional techniques were also studied for nisin purification by Forestier et al. [16], who purified nisin by ultrafiltration from a citrate milk supernatant. Using a 10 kDa polyether sulfone membrane, ultrafiltration allowed for a nisin purification factor of 4.0 and a 100% yield.

While displaying comparable to or better nisin purification factors and yields compared with common and other techniques (Table 4), ED presents the advantages of being an eco-friendly process with a low energy consumption and using no chemical solvents [36]. Also, ED is a versatile process that can be implemented in industrial downstream processing compared with the other techniques, which present high costs [58].

## 4. Conclusions

In this work, the purification of nisin by ED and its efficiency in terms of its great fold purification compared with commonly used techniques was demonstrated. To the best of our knowledge, no study has reported on the use of ED for nisin purification from a CFS after production when using whey permeate as a low-cost medium. Prior to nisin purification by ED, an optimal ASS level of 40% was determined, with a purification factor of 73.8 and an activity yield of 60% (compared with 34.5 with a 50% activity yield at a 60% ASS level). This allowed us to perform nisin purification by ED coupled with salting out. The coupling of salting out and ED produced a highly concentrated nisin fraction from a CFS after fermentation on whey permeate. Indeed, a purification factor of 21.8 with a nisin activity yield of 70% were determined when comparing the specific activity of the initial CFS to the solution after ED. A nisin concentration factor of 156-fold was confirmed by UPLC-MS. These purification factors and yields were, to our knowledge, the best results demonstrated for nisin purification when compared with commonly used techniques. The greater performance of the nisin purification by salting out at a 40% ASS level can be attributed to the competition induced by ammonium sulfate salts, affecting the proteins’ access to water molecules and the importance of the proteins’ characteristic. Moreover, no scaling phenomenon appeared during the nisin purification, since no differences were observed for IEM characterization after and before nisin demineralization.

**Figure 5:**
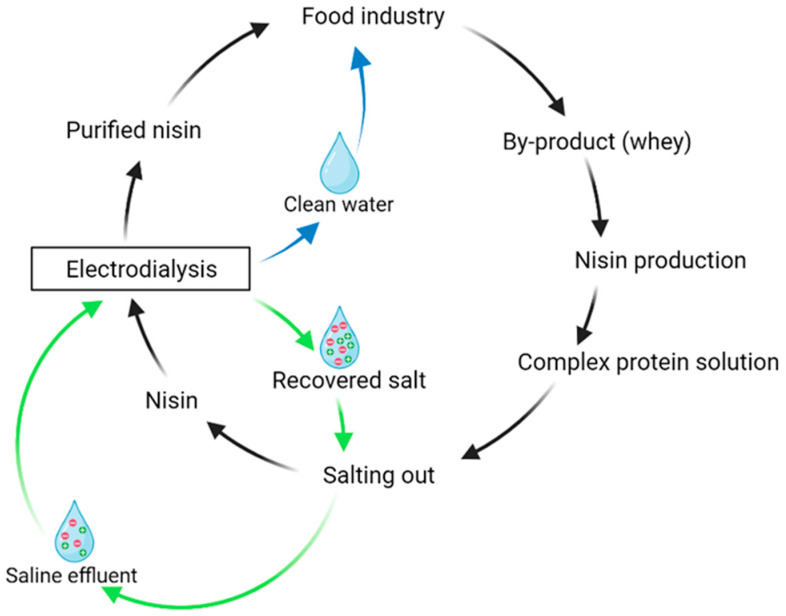
Nisin purification by ED after salting out and further treatment of saline effluents produced in the context of a circular economy.

These results are, moreover, highlighted by the advantages of ED. Indeed, ED is a more eco-friendly technique, using no chemical solvents and having a lower energy consumption compared with other methods employed for purification or desalinization [36,59]. Its capacity to process saline effluents could also be of interest. As illustrated in Figure 5, a circular economy scheme could be implemented initially from the production of nisin, using by-products generated by the food industry such as whey. The use of whey or other by-products would allow for the recycling of effluents produced in the food industry into fermentation broths and to the reduction of nisin production costs, one of the main current hurdles for its production. In addition, the circular economy concept would be reflected by (1) the use of ED in the purification of nisin from a food by-product to produce a purified nisin with further applications as a food preservative within the same food industry and (2) considering ED’s capacity to treat saline effluents produced during the salting out step. Among the different methods used for saline effluent treatment such as thermal methods, biological methods or membrane-based methods, ED could allow for the recycling of salts by reusing the concentrated salt solution for the salt precipitation step in the nisin production scheme. The implementation of ED in a circular economy, from nisin production to the treatments and recovery of salts from saline effluents, is a novel perspective, proposed for the first time here. Indeed, the use of ED for the treatment of high-salt effluent and wastewater was already reported by Bazinet et al. (2020) and Chen et al. (2020) [59,60], but investigations in order to implement saline effluent treatment with ED in this scheme could be of interest. Further investigations regarding production costs and life cycle assessments for the eco-circular scheme would be necessary to confirm its viability.

## Figures and Tables

**Table 1:** Protein content, antimicrobial activity, fold purification and antimicrobial activity yield of nisin after salting out of CFS at different conditions of ammonium sulfate saturation levels tested.

Salt Saturation	Volume (mL)	Mass of Protein Recovered (mg) *	Total Activity (AU)	Antimicrobial Activity Yield (%)	Specific Activity (AU/mg of Protein)	Fold Purification
CFS	200	725.1 ± 120.1 ^a^	51200 ^a^	100 ^a^	72.0 ± 12.7 ^c^	1 ^a^
20%	30	n/d ^c^	2240 ^a^	4.4 ^a^	n/d	n/d ^a^
40%	30	5.9 ± 1.2 ^c^	30720 ^a^	60 ^a^	5310.4 ± 1007.1 ^a^	73.8^a^
60%	30	10.3 ± 1.7 ^b^	25600 ^a^	50 ^a^	2481.6 ± 827.2 ^b^	34.5 ^a^

* Protein content was determined on the total volume of CFS used for salting out and the resolubilized pellet after salting out. Mean ± standard deviation. Data with same letters on the same column are not significantly different at *p* < 0.05 (ANOVA). n/d: could not be determined.

**Table 2:** Membrane thickness and conductivity before and after electrodialysis treatment.

Membrane	Thickness (mm)		Conductivity (mS/cm)	
	Before	After	Before	After
CEM 1	0.138 ± 0.008 ^a,^*^,A,^**	0.138 ± 0.006 ^a,A^	8.47 ± 0.29 ^a,A^	7.73 ± 0.47 ^a,A^
CEM 2	0.138 ± 0.006 ^a,A^	0.143 ± 0.003 ^a,A^	8.40 ± 0.30 ^a,A^	7.93 ± 0.21 ^a,A^
CEM 3	0.143 ± 0.004 ^a,A^	0.140 ± 0.006 ^a,A^	8.63 ± 0.75 ^a,A^	8.03 ± 0.21 ^a,A^
AEM 1	0.145 ± 0.009 ^a,A^	0.138 ± 0.006 ^a,A^	5.90 ± 0.62 ^a,B^	5.27 ± 0.35 ^a,B^
AEM 2	0.144 ± 0.010 ^a,A^	0.140 ± 0.006 ^a,A^	5.43 ± 0.35 ^a,B^	5.23 ± 0.21 ^a,B^

Mean ± standard deviation. * Values with the same lowercased letter for the same line before and after, for the same analysis (thickness or conductivity), are not significantly different at *p* < 0.05 (*t*-test). ** Values with the same capital letter for the same column, for the same analysis (thickness or conductivity), are not significantly different at *p* < 0.05 (*t*-test).

**Table 3:** Parameters assessed on initial CFS, after salting out and after ED.

Samples	Volume (mL)	Protein Content (mg)	Total Activity (AU)	Specific Activity (AU/mg of Protein)	Activity Yield (%)	Fold Purification
CFS (initial)	2000	6760.0 ± 383.0	512,000	75.9 ± 4.4	100	1.0
After salting out	350	n/d	358,400	n/d	70	n/d
After ED	350	219.7 ± 29.7	358,400	1652.7 ± 236.8	70	21.8

n/d: under the limit of detection or sensitivity of the method.

**Table 4:** Comparison of techniques used for nisin purification and their respective purification folds and nisin activity yields.

Technique	Source of Nisin	Fold Purification	Nisin Activity Yields (%)	Reference
Electrodialysis with salting out	Crude extract	21.8	70	Present study
Electrodialysis	2.5% nisin solution	1.9	/	[33]
Ammonium sulfate	Crude extract	3.8	94	[25]
	Crude extract	2.5	62	[24]
	Crude extract	5.1	98	[31]
Cation exchange chromatography	Crude extract	31	20	[20]
Expanded-bed IEC	Crude extract	31	90	[9]
Immunoaffinity chromatography	Crude extract	10	72	[23]
Hydrophobic interaction chromatography with salting out	Crude extract	10.9	50.8	[24]
Methanol	2.5% nisin solution	5.3	91	[56]
	2.5% nisin solution	6.0	52.4	[57]
Ethanol	2.5% nisin solution	5.5	85	[56]
	2.5% nisin solution	1.9	/	[57]
Chloroform	Crude extract	37.4	24	[20,26]
Ultrafiltration	Crude extract	4.01	100	[16]

## Data Availability

Data are contained within the article.
